# Targeting androgen receptor as a new potential therapeutic approach to battle tobacco carcinogens-induced non-small cell lung cancer

**DOI:** 10.1186/1479-5876-10-S2-A8

**Published:** 2012-10-17

**Authors:** Shauh-Der Yeh, Pan-Chyr Yang, Hsuan-Hsuan Lu, Chawnshang Chang, Cheng-Wen Wu

**Affiliations:** 1 Department of Urology and Center of Excellence for Cancer Research, Taipei Medical University and Hospital, Taipei, 110, Taiwan; 2 Institute of Biomedical Sciences, Academia Sinica, Taipei, 115, Taiwan; 3 Department of Pathology, University of Rochester, Rochester, NY 14642, USA

## Background

Lung cancer is the leading cause of cancer death worldwide. Non-small cell lung cancer (NSCLC) accounts for the majority of lung cancer. The incidence and prognosis in NSCLC demonstrates gender difference [1,2]; therefore, sex steroids and/or their receptors may play important roles during lung tumorigenesis and cancer progression. In our previous investigation, cell proliferation, migration, invasion, and tumor formation were inhibited by shRNA interference of androgen receptor (AR) in non-small cell lung cancer (NSCLC) cells lines. The expressions of cyclin D1 also decreased to less than 50% after AR knockdown. However, the roles of androgen receptor in treatment of NSCLC are still controversial.

## Materials and methods

To validate therapeutic effects of targeting androgen receptor on NSCLC, initially we administered 8 doses of tobacco carcinogens, 4-(methylnitrosamino)-1-(3-pyridyl)-1-butanone (NNK) and benzo[a]pyrene (BaP), to induce lung tumor growth in female A/JB6.129-*Ar^lox^* Tg(Mx1-cre)1Cgn mice when they were 5 weeks old. In the next step, targeted *Ar* gene disruption was induced with 6 doses of polyinosinic: polycytidylic acid (polyI:C) intraperitoneal injection in Mx1-cre+ mice when they were 26 weeks old. We also performed 6 doses of normal saline injection to NNK+BaP-treated female A/JB6.129-*Ar^lox^* Tg(Mx1-cre)1Cgn mice in the same time point for the control group. Finally, all mice were sacrificed in 31 weeks old and total lung nodules and tumor larger than 1 mm in diameter were calculated under dissection microscope.

## Results

In immunohistochemical studies, AR expression in lung was deficient in Mx1-cre+ mice after polyI:C treatment. Pulmonary expression of cyclin D1 was also suppressed in polyI:C treated Mx1-cre+ mice. The number of total nodules in bilateral lungs from polyI:C treated Mx1-cre+ mice (n=8) was 7.375 ± 5.476 (mean ± S.E.). In comparison, the total number of lung nodules from normal saline treated Mx1-cre+ mice (n=8) was 14.375 ± 7.269 (p= 0.0456, 95% CIs on the mean = 9.235 to 19.515). The number of large nodules (>1 mm in diameter) in bilateral lungs was 1.375 ± 1.188 in polyI:C treated Mx1-cre+ mice and 6.25 ± 4.464 in normal saline treated Mx1-cre+ control mice (p= 0.00492, 95% CIs on the mean = 3.093 to 9.407). Deficient AR expression through inducible disruption of *Ar* gene could reduce lung tumor multiplicity and further inhibit tumor progression (decrease tumor volume) in tobacco carcinogens-induced lung tumorigenesis model.

## Conclusions

Our data indicate that androgen receptor warrants consideration as a novel therapeutic target for NSCLC in a clinical lung cancer treatment trial.
